# Effect of Storage Period on the Changes of Odorous Compound Concentrations and Bacterial Ecology for Identifying the Cause of Odor Production from Pig Slurry

**DOI:** 10.1371/journal.pone.0162714

**Published:** 2016-09-19

**Authors:** Ok Hwa Hwang, Sung Back Cho, Deug Woo Han, Sang Ryoung Lee, Jeong Hoon Kwag, Sung Kwon Park

**Affiliations:** 1 National Institute of Animal Science, Rural Development Administration, Wanju-Gun, Jeollabuk-Do, Republic of Korea; 2 Department of Food Science and Technology, Sejong University, Seoul, Republic of Korea; Duke University, UNITED STATES

## Abstract

Odor from buildings where pigs are housed is generated by anaerobic fermentation of undigested materials in pig slurry stored for several weeks in pit. The objective of this study was to investigate the effect of storage period on the level of odorous compounds in pig slurry and on its bacterial community. A slurry sample (15 L) was taken from the pit of a finisher pig building and incubated in acryl chambers for six- weeks. Slurry for analysis was sampled every two-week. Levels of odorous compounds in the slurry sample were drastically changed after two weeks of storage period; levels of phenols and short chain fatty acids (SCFAs) were decreased (P<0.05), whereas indoles and branched-chain fatty acids (BCFAs) were increased (P<0.05). Among dominant bacteria, *Bacteroides* and *Porphyromonadacese_uc_g* revealed a strong positive correlation with the levels of phenols and SCFAs. Populations of *AC160630_g*, *Acholeplasmatales_uc_g*, *Mollicutes_uc_g* and *Cloacamonas_f_uc_g* positively correlated with indole and BCFAs content. Taken together, levels of odorous compounds were increased after two weeks of storage, possibly because of changes in the predominant bacterial groups to those that use protein as a carbon source in the hypo-carbohydrate conditions.

## Introduction

Large amounts of pig slurry are generated by intensive animal farming and industrial livestock production (factory farming); in South Korea, the amount increased from 4,370 million tons in 2009 to 4,724 million tons in 2013 [1]. Swine farming accounts for more than 54% of civil complaints about odor from livestock facilities in South Korea [2]. Odor emissions from pig farms are mainly affected by the type of pig house; in South Korea, 76% of pig houses are built with an open ventilation system and 52% with a slurry storage system [2]. To cut down livestock odor, the Korean government enacted an offensive odor control law [3] and restricted the zones where livestock buildings are constructed [4]. Odor control is a prerequisite for successful and sustainable pig production coexisting with nearby residents [5].

Pig slurry is usually stored for a couple of weeks to months inside a pit under the pig building before being cleaned out. During this storage period, anaerobic fermentation triggered by microbes using the undigested nutrients and endogenous materials in the slurry is the main cause of malodor generation [6]. Surface crust and sediment layer formation is another cause of odor [7]. More than 200 chemical compounds are known to be related to livestock odor, but only several compounds are contributed to the offensive odor [8–10]. Recently, researchers identified amines, ammonia, volatile fatty acids (VFAs), phenols and indoles as key odorants in feedlot manure [5, 9–14].

Most of the odor-causing materials are produced by protein degradation. If carbohydrates are scarce in pig slurry during the storage period, protein becomes a primary source of fermentable carbon [15]. The nutritional composition of slurry also modulates the bacterial community and its end-metabolites [16]. A few studies have investigated the causes of odor, indicating that there is a correlation between the composition of the bacterial community and odorous compounds [17, 18]. However, these correlations are hard to interpret because of the rapidly changing environment of pig production, including feed ingredients and the physical condition of the pigs.

The objectives of this study were to identify the cause of odor from pig houses with regard to the effect of the slurry storage period on the changes in concentration of odorous compounds and the composition of bacterial communities.

## Materials and Methods

### Experimental design and slurry collection

Fresh slurry was collected from the pit under a pen housing finisher pigs [total of 60 {(Landrace × Yorkshire) × Duroc}] with body weight (BW) of 80~110 kg in National Institute of Animal Science, Wanju-Gun, Jeollabuk-Do (GPS: 35°49´26.7"N, 127°03´31.6"E). The finisher pigs were fed a basal diet formulated according to the Korean Feeding Standard [19]. Fifteen liters of slurry were incubated in an acryl chamber for 6 weeks at room temperature (20~25°C). Air was continuously supplied to the upper compartment of the chamber with 15 mL/min velocity [20]. Slurry was sampled every 2 weeks for analysis of odorous compounds and the bacterial community. Odorous compounds including VFAs [short chain fatty acids (SCFAs) and branched-chain fatty acids (BCFAs)], phenols and indoles were measured by gas chromatography (GC). The bacterial community was analyzed by pyrosequencing using the 454 FLX Titanium System (Roche, Pleasanton, CA, USA).

### Odorous compound analysis

#### Volatile Fatty Acids

Five milliliters of slurry were mixed with 1 mL of 25% meta-phosphoric acid solution (Sigma-Aldrich, St. Louis, MO, USA) and 0.05 mL of saturated mercury (II) chloride solution (Sigma-Aldrich, St. Louis, MO, USA) in a 15 mL plastic tube. The mixed solution was then centrifuged at 3,134 × *ɡ* for 20 min at 20°C. One milliliter of supernatant was subsequently centrifuged for 10 min at 13,800 × *ɡ* and filtered through a 0.2 μm filter (Whatman, Uppsala, Sweden). Filtrates were transferred to 2.0 mL GC vials (Agilent, Santa Clara, CA, USA). The concentration of VFAs was analyzed using a GC (6890N, Agilent, Santa Clara, CA, USA) equipped with a HP-INNOWax column (30 m × 0.25 mm × 0.25 μm; Agilent, Santa Clara, CA, USA) and a flame ionization detector (FID). The sample injection volume was 0.2 μL with a 10:1 split ratio. The oven temperature was initially temperature of 80°C for 2 min, increasing to 120°C at 20°C/min, then to 205°C at 10°C/min, and finally held at 205°C for 2 min. The injection and detection ports were maintained at 250°C.

#### Phenols and indoles

Slurry samples were centrifuged at 3,134 × *ɡ* for 20 min at 20°C, and then 4 mL of supernatant was mixed with 4 mL of chloroform (Merck, Darmstadt, Germany) and 60 μL of 4M sodium hydroxide solution (Sigma-Aldrich, St. Louis, MO, USA) in a 20 mL glass vial. The mixture was centrifuged at 3,134 × *ɡ* for 20 min at 20°C, and the chloroform layer was transferred to a 2.0 mL GC vial (Agilent, Santa Clara, CA, USA). Phenols and indoles were analyzed using a GC (6890N, Agilent, Santa Clara, CA, USA) equipped with a DB-1 column (30 m × 0.25 mm × 0.25 μm, Agilent, Santa Clara, CA, USA) and a FID. The sample injection volume was 2.0 μL with a 5:1 split ratio. The oven temperature was initially 40°C for 5 min, increasing to 230°C at 10°C/min, which was then held at 230°C for 2 min. The injection and detection ports were maintained at 250°C.

### Bacterial community analysis

#### PCR amplification for bar-coded pyrosequencing

Total genomic DNA from slurry was extracted using a Fast-DNA Spin Kit (MP Bio, Santa Ana, CA, USA) according to the manufacturer’s instructions. Humic acid interferes with PCR amplification was removed using a Power-Clean DNA Clean-Up Kit (MP Bio, Santa Ana, CA, USA). Bacterial 16S ribosomal RNA (16S rRNA) genes around 500–700 bp long containing V1 to V3 of the variable region were amplified using primer set 27F (5'-adaptor 2-AC-GAG TTT GAT CMT GGC TCA G-3') and 518R (5'-adaptor 1-AC-X-WTT ACC GCG GCT GCT GG-3') where “X” denotes unique 7 to 11 bar-code sequences inserted between the 454 Life Sciences adaptor A sequence and the common linker, AC [21]. PCR amplification conditions were one cycle of 95°C for 5 min, followed by 30 cycles of 95°C for 30 sec, 55°C for 30 sec and 72°C for 30 sec, and finally one cycle of 72°C for 7 min.

#### Pyrosequencing and data analysis

Pyrosequencing was performed by ChunLab (Seoul, Korea) using a 454 FLX Titanium System (Roche, Pleasanton, CA, USA). Sequencing reads were assigned to specific samples based on their unique barcodes. Then barcode, linker and PCR primer sequences at both ends were removed from the original sequencing reads. The final pyrosequencing reads for subsequent analysis were selected by a filtering process including only reads containing >300 base pairs and an average quality score >25. Taxonomic assignment of the bacterial high quality reads was performed using the EzTaxon-e database [22] and a robust global pairwise sequencing alignment, coupled with the BLAST search tool (http://www.ncbi.nlm.nih.gov/BLAST). Sequences that could be matched to the EzTaxon-e database at the species level (>97%) were subjected to a secondary process to check for chimeric sequences using the UCHIME program [23]. Operational taxonomic units (OTUs) were generated using the CD-HIT program at a 97% similarity level. The Shannon-Weaver diversity index, Chao1 richness index and Goods library coverage were calculated using the Mothur package [24].

### Statistical analysis

All experimental data including those concerning odorous compounds and bacterial communities were subjected to analysis of variance for a completely randomized design using the general linear model procedures of SAS software [25]. Significant differences among treatments were compared with Duncan’s multiple range tests [26]. Hierarchical clustering, principal component analysis (PCA) and principal coordinate analysis (PCoA) were performed to examine any grouping dependent upon the storage period. Pearson’s correlation coefficient was used to determine the link between bacterial genera and odorous compounds. Multiple statistical comparisons were evaluated using the data for odorous compounds and the bacterial community in the SAS program [25]. The threshold for significance was P<0.05 for all measured variables.

## Results

### Concentration of odorous compounds in pig slurry by storage period

The effects of a storage period up to 6 weeks on phenols, indoles and VFAs concentration in pig slurry are shown in Table 1. The concentrations of odorous compounds were dramatically changed at 2 weeks-post storage period. Comparing 2 weeks storage to 4 weeks, phenols levels were decreased (P<0.05) by 18% (from 187.40 to 153.79 mg/L), and indoles levels were increased (P<0.05) by 44% (from 4.11 to 7.29 mg/L). SCFAs levels were decreased (P<0.05) by 18% (from 9,185 to 7,516 mg/L), and BCFAs levels were increased (P<0.05) by 24% (from 1,167 to 1,444 mg/L).

**Table 1:** Concentration of odorous compounds in pig slurry by storage period.

Items (mg/L)	Storage period (weeks)				SEM^5^	SD^6^
	0	2	4	6		
Phenol	16.02^a^	13.93^b^	11.28^c^	9.91^d^	0.36	2.50
p-Cresol	199.03^a^	173.47^b^	142.51^c^	138.57^c^	3.84	26.57
Indole	0.57^c^	0.84^b^	1.55^a^	1.60^a^	0.07	0.47
Skatole	3.21^b^	3.27^b^	5.74^a^	5.95^a^	0.19	1.35
Phenols^1^	215.06^a^	187.40^b^	153.79^c^	148.47^c^	4.19	29.04
Indoles^2^	3.78^c^	4.11^b^	7.29^a^	7.54^a^	0.26	1.81
Acetic acid	7,002^a^	6,480^a^	5,391^b^	5,831^b^	133.45	924.54
Propionic acid	2,181^a^	1,858^b^	1,358^c^	1,424^c^	57.40	397.64
Butyric acid	1,239^a^	847^b^	767^b^^c^	718^c^	33.69	233.44
Iso-Butyric acid	610^b^	603^b^	731^a^	772^a^	15.67	108.60
Iso-Valeric acid	562^b^	565^b^	714^a^	773^a^	17.29	119.82
SCFAs^3^	10,423^a^	9,185^b^	7,516^c^	7,973^c^	219.02	1,517.4
BCFAs^4^	1,172^b^	1,167^b^	1,444^a^	1,544^a^	32.88	227.79
pH	8.11^d^	8.39^b^	8.28^c^	9.08^a^	0.10	0.38

^1^ Phenols = phenol + p-cresol.

^2^ Indoles = indole + skatole.

^3^ SCFAs (Short chain fatty acids) = acetic acid + propionic acid + butyric acid [27].

^4^ BCFAs (Branched-chain fatty acids) = iso-butyric acid + iso-valeric acid.

^5^ SEM: Standard errors of the means.

^6^ SD: Standard deviation.

^a, b, c, d^ Figures with different superscripts within the same row are significantly different (P<0.05).

### Changes in bacterial community composition in pig slurry by storage period

#### Pyrosequencing data

Changes in the bacterial community structure during 6 weeks slurry storage period were analyzed by the multiplex bar-coded pyrosequencing technique based on 16S rRNA gene sequences (Table 2). After removal of low quality reads from the sequencing data, the total numbers of reads were 6,078 (0 weeks), 4,141 (2 weeks), 3,658 (4 weeks) and 4,000 (6 weeks), respectively. Although the number of bacterial reads was not significantly different after 2 weeks of storage, the number of OTUs, the Shannon-Weaver index and Chao 1 index were decreased (P<0.05) at 2 weeks-post storage.

**Table 2:** Pyrosequencing data in bacterial community composition in pig slurry by storage period.

Items	Storage period (weeks)				SEM^3^	SD^4^
	0	2	4	6		
No. of total reads	7,636^a^	5,391^b^	4,482^b^	4,545^b^	420.99	1,458.36
No. of high-quality reads	6,078^a^	4,141^b^	3,658^b^	4,000^b^	317.36	1,099.37
Aver. read length (bp)	504^b^	504^a^^b^	503^b^	505^a^	0.28	0.98
OTUs^1^	1,466^a^	999^b^	738^c^	605^c^	104.11	360.66
Shannon-Weaver index(H’)^2^	5.63^a^	5.36^a^	4.78^b^	4.29^c^	0.16	0.56
Chao1 index^2^	3,491^a^	2,433^b^	1,841^b^^c^	1,335^c^	245.11	880.26
Goods library coverage	0.88^c^	0.88^b^^c^	0.90^b^	0.92^a^	0.01	0.02

^1^ OTUs: Operational Taxonomic Units.

^2^ Shannon-Weaver (diversity index), Chao1 (richness index) and Goods library coverage were calculated using the Mothur package.

^3^ SEM: Standard errors of the means.

^4^ SD: Standard deviation.

^a, b, c^ Figures with different superscripts within the same row are significantly different (P<0.05).

#### Bacterial taxonomic composition

**Fig 1:**
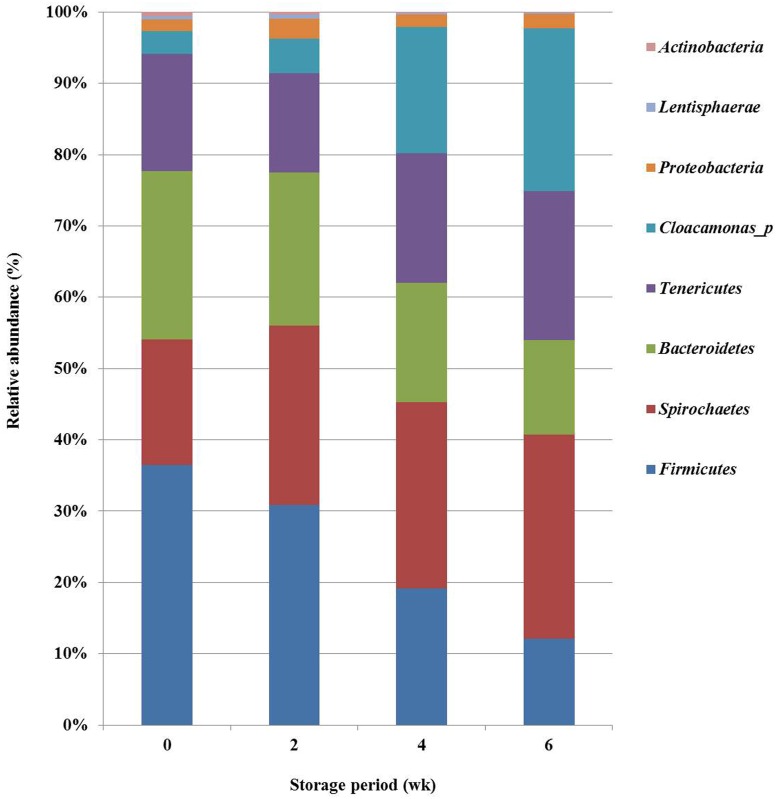
Bacterial taxonomic composition of phylum level in pig slurry by storage period. Sequences were classified using the EzTaxon-e database with an 80% confidence threshold. “wk” is an abbreviation to weeks.

Taxonomic pyrosequencing profiles of bacterial communities in pig slurry are shown in Figs 1 (phylum level) and 2 (genus level). The eight major phyla among a total of 27 included *Firmicutes*, *Spirochaetes*, *Bacteroidetes*, *Tenericutes*, *Cloacamonas_p*, *Proteobacteria*, *Lentisphaerae* and *Actinobacteria* (Fig 1). The relative abundance of *Firmicutes* and *Lentisphaerae* was reduced (P<0.05) after 2 weeks of storage. *Bacteroidetes* consistently decreased (P<0.05) during the 6 weeks storage period. However, *Cloacamonas_p* was drastically increased (P<0.05) after 2 weeks of storage.

**Fig 2:**
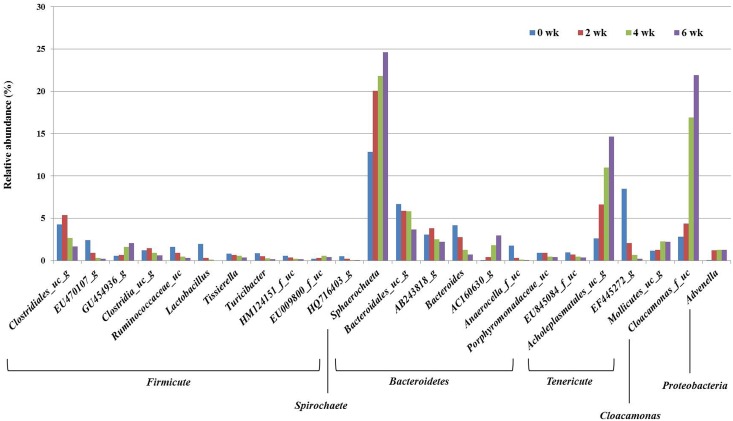
Bacterial taxonomic composition of genus level in pig slurry by storage period. Bacterial genus were classified at a cut off level of >0.5% relative abundance and grouped according to phylum level. “wk” is an abbreviation to weeks.

At the genus level (Fig 2), a total of 514 bacterial genera were represented in the sequences; they could be classified by phylum as: 253 *Firmicutes*, 70 *Proteobacteria*, 64 *Bacteroidetes*, 29 *Actinobacteria*, 23 *Tenericutes*, 21 *Lentisphaerae*, 7 *Cloacamonas_p* and 7 *Spirochaetes*. Altogether, there were 305 Gram-positive bacterial genera and 169 Gram-negative genera. Among the dominant genera, *Clostridiales_uc_g*, *EU470107_g*, *Ruminococcaceae_uc*, *Lactobacillus*, *Turicibacter* and *HQ716403_g* of the phylum *Firmicutes*, and *Bacteroidales_uc_g*, *Bacteroides*, *Anaerocella_f_uc* and *Porphyromonadaceae_uc* of the phylum *Bacteroidetes* were decreased (P<0.05) for 6 weeks of storage. Within these genera, *Clostridiales_uc_g*, *Bacteroides* and *Porphyromonadaceae_uc* were decreased (P<0.05) by 2 weeks. *Sphaerochaeta* of the phylum *Spirochaetes*, *Cloacamonas_f_uc* of *Cloacaminas_p*, *GU454936_g* of *Firmicutes*, *AC160630_g* of *Bacteroidetes*, *Acholeplasmatales_uc_g* and *Mollicutes_uc_g* of *Tenericutes*, and *Advenella* of *Proteobacteria* were increased (P<0.05) for 6 weeks of storage. Within these genera, *Cloacamonas_f_uc*, *GU454936_g*, *AC160630_g*, *Acholeplasmatales_uc_g* and *Mollicutes_uc_g* were drastically increased (P<0.05) after 2 weeks.

### Statistical comparison of odorous compounds and bacterial community compositions in pig slurry by storage period

**Fig 3:**
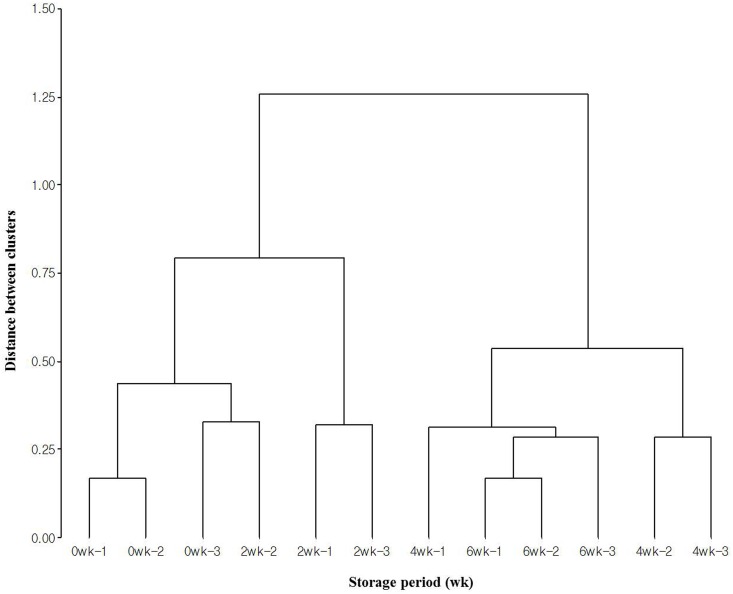
Hierarchical clustering result showing the group by storage period. The clustering data was constructed using the concentrations of odorous compounds and the values of relative abundances in bacterial genus level. The scale bars represent the distance between clusters. “wk” is an abbreviation to weeks.

**Fig 4:**
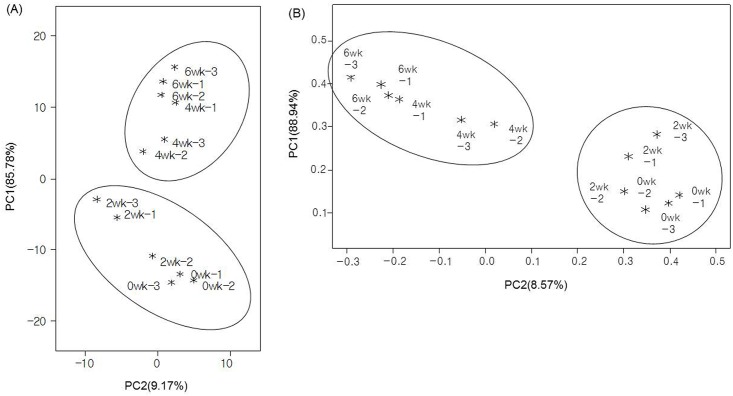
(A) Principal component analysis (PCA) and (B) principal coordinate analysis (PCoA) plot result showing the group by storage period. These data were created using the concentrations of odorous compounds and the values of relative abundances in bacterial genus level. “wk” is an abbreviation to weeks.

Changes in the bacterial compositions and concentrations of odorous compounds during the 6 weeks storage were graphically summarized using hierarchical clustering, PCA and PCoA in Figs 3 and 4. Two clusters were divided into one group of 0 and 2 weeks and another group of 4 and 6 weeks.

### Correlation between odorous compound production and dynamics of the bacterial community

The interrelationships of various bacterial genera associated with odorous compounds analyzed in the current study is shown in Table 3. Compared with other bacterial genera, *Bacteroides*, *Porphyromonadaceae_uc_g*, *AC160630_g*, *Acholeplasmatales_uc_g*, *Mollicutes_uc_g* and *Cloacamonas_f_uc* showed relatively greater (P<0.05) correlation coefficient values with odorous compounds. There was a positive correlation between phenol, p-cresol, acetic acid, propionic acid and butyric acid with *Bacteroides* and *Porphyromonadaceae_uc_g*, whereas there was a negative correlation with *AC160630_g*, *Acholeplasmatales_uc_g*, *Mollicutes_uc_g* and *Cloacamonas_f_uc*. Indole, skatole, iso-butyric acid and iso-valeric acid were shown to have the opposite correlations with these genera.

**Table 3:** Correlation coefficient (R) values for the relative abundance of bacterial genera with the odorous compounds in pig slurry.

Items	Odorous compounds								
	Phenol	p-Cresol	Indole	Skatole	Acetic acid	Propionic acid	Butyric acid	I-Butyric acid	I-Valeric acid
Genus									
* Clostridiales_uc_g*	0.70	0.64	-0.72	-0.83	0.65	0.68	0.49	-0.77	-0.81
* EU470107_g*	0.84	0.85	-0.84	-0.77	0.76	0.84	0.89	-0.71	-0.76
* GU454936_g*	-0.89	-0.84	0.89	0.95	-0.85	-0.88	-0.76	0.81	0.88
* Clostridia_uc_g*	0.59	0.54	-0.59	-0.72	0.56	0.57	0.41	-0.62	-0.66
* Ruminococcaceae_uc*	0.87	0.87	-0.87	-0.83	0.77	0.85	0.86	-0.77	-0.82
* Lactobacillus*	0.75	0.77	-0.75	-0.64	0.76	0.80	0.89	-0.49	-0.57
* Tissierella*	0.82	0.78	-0.84	-0.84	0.74	0.81	0.80	-0.69	-0.77
* Turicibacter*	0.81	0.80	-0.82	-0.78	0.78	0.83	0.85	-0.66	-0.73
* HM124151_f_uc*	0.86	0.87	-0.81	-0.74	0.72	079	0.80	-0.72	-0.77
* EU009800_f_uc*	-0.70	-0.75	0.77	0.77	-0.84	-0.81	-0.67	0.69	0.70
* HQ716403_g*	0.85	0.87	-0.84	-0.77	0.76	0.84	0.87	-0.74	-0.79
* Sphaerochaeta*	-0.77	-0.76	0.75	0.70	-0.63	-0.73	-0.80	0.66	0.72
* Bacteroidales_uc_g*	0.81	0.73	-0.73	-0.67	0.66	0.71	0.76	-0.47	-0.59
* AB243818_g*	0.61	0.57	-0.65	-0.76	0.63	0.62	0.39	-0.65	-0.68
* Bacteroides*	0.96	0.96	-0.93	-0.88	0.88	0.93	0.91	-0.77	-0.85
* AC160630_g*	-0.95	-0.91	0.95	0.94	-0.86	-0.91	-0.83	0.80	0.88
* Anaerocella_f_uc*	0.69	0.72	-0.68	-0.60	0.62	0.69	0.79	-0.53	-0.59
* Porphyromonadaceae_uc*	0.82	0.81	-0.85	-0.90	0.71	0.78	0.65	-0.93	-0.94
* EU845084_f_uc*	0.88	0.87	-0.86	-0.82	0.80	0.85	0.84	-0.73	-0.80
* Acholeplasmatales_uc_g*	-0.96	-0.93	0.95	0.91	-0.84	-0.91	-0.89	0.82	0.89
* EF445272_g*	0.82	0.84	-0.79	-0.69	0.68	0.78	0.87	-0.67	-0.72
* Mollicutes_uc_g*	-0.88	-0.88	0.91	0.93	-0.87	-0.90	-0.75	0.86	0.90
* Cloacamonas_f_uc*	-0.90	-0.87	0.91	0.97	-0.85	-0.89	-0.78	0.85	0.91
* Advenella*	-0.70	-0.72	0.69	0.54	-0.62	-0.69	-0.80	0.50	0.56

Correlation among parameters analyzed using Pearson correlation and stepwise multiple linear regression models (P<0.05).

## Discussion

The generation of odorous compounds from pig house is contributed to bacterial fermentation within the gastrointestinal tract of the pigs and the slurry in pit under the floor of the pig pen. Bacteria utilize undigested dietary materials, endogenous compounds and dead bacterial cells in slurry [17], and thereby generate various odorous compounds. Nitrogen is the major precursor of odorous compounds [28]. Gut bacteria obtain energy for maintaining homeostasis and growth from carbohydrates, and for building blocks from proteins included in the pig diet and endogenous materials. Availability of carbohydrate is very low while pig slurry is stored in the pit, thus protein becomes the main energy source for maintaining bacterial homeostasis [29]. In these circumstances, generation of offensive odorous compounds is augmented during bacteria-mediated amino acid metabolism [30]. This study was performed to identify the various odorous compounds generated from pig slurry stored for different periods, and their correlation with the bacterial taxonomic composition.

Phenols and indoles are produced during bacterial metabolism of tyrosine and tryptophan, respectively, in stored manure [27]. They are absorbed via epithelial cells of the large intestine, conjugated with glucuronic acid and detoxified to glucuronides in the liver. Glucuronides are excreted via urine and then hydrolyzed to phenols and indoles by fecal β–glucuronidase [31]. For this reason, combining the feces and urine results in raising the content of free phenolic or indolic compounds. They have a low odor detection threshold and relatively high in odor intensity and nuisance [28, 32]. The odor detection threshold is the lowest concentration of a certain odorous compound that is perceivable with the human sense of smell [33]. The detection thresholds for phenol, p-cresol, indole and skatole are 0.11, 0.00186, 0.0000316 and 0.000562 ppm, respectively [11, 34–36]. Among the volatile organic compounds (VOCs) emitted from pig buildings, manure storage sites and land application, p-cresol, indole and skatole account for more than 90% of the odor activity value. In particular, p-cresol is most responsible for the overall odor impact from the VOCs emission [37–39].

In this study, dramatic changes in the contents of phenols and indoles were detected at 2 weeks in stored slurry. Results from others have shown that a decrease in the concentration and emission rate of phenols started at about 36-day, but the indoles level was remained constant over 71-day storage period [40]. Ziemer, Kerr [41] reported that increased level of phenols and decreased level of indoles were detected after 8 weeks of storage, which correlated with a decrease in the pH. Phenols are formed at low pH, but indoles are accumulated at high pH [42–44]. Therefore, decreased level of phenols and consistently high indoles level might relate to increased pH during slurry storage in our current study. In addition, phenol and p-cresol are degraded by aerobic bacteria [45], and phenol is also decomposed to CO_2_ by anaerobic bacteria in stored pig waste [46]. Bacterial decomposition is associated with bacterial growth when carbohydrate sources become limited as the storage period passes.

SCFAs are a product of carbohydrate fermentation, whereas BCFAs are a product of protein fermentation [27]. SCFAs are an important metabolic fuel for bacterial growth. SCFAs are absorbed into animal blood, transported to organs and tissues, and subsequently metabolized to energy sources for gut bacteria [27]. Decreased levels of SCFAs and increased BCFAs levels reflects a decrease in energy source availability in pig manure [40]. In the present study, SCFAs levels were decreased, but BCFAs levels increased in slurry stored for 6 weeks. VFAs concentrations dramatically changed after 2 weeks of storage. Miller and Varel [47] reported that a decrease in SCFAs (13%) and an increase in BCFAs (39%) occurred after 37 days of storage of pig slurry. Levels of VFAs are also affected by slurry pH; the BCFAs levels were increased by 67% at high pH (5.5 compared with 6.8) [48].

Analyzing the relationship between bacteria and their biotopes is an important step to understanding the mechanism of accumulation of odorous compounds produced by bacterial fermentation. The bacterial community in pig slurry was previously characterized by culture methods [49–51]. Recently, multiplex bar-coded pyrosequencing techniques based on 16S rRNA genes have been used to identify members of the bacterial community comprising as little as 1% of the total population [7, 17, 52].

According to previous study [53], the bacterial community changed in pig slurry stored for 3 weeks; the number and diversity of bacteria decreased. In the present study (Fig 1) the relative abundance of *Firmicutes*, *Bacteroidetes* and *Lentisphaerae* decreased but *Cloacamonas_p* increased after 2 weeks storage of pig slurry. *Firmicutes* and *Bacteroidetes* constituted the majority of gut bacteria. Bacterial strains belonging to these two phyla are used as a phylogenetic markers because their relative abundance is easily influenced by fermentation conditions [54]. At the genus level, *Clostridiales_uc_g*, *Bacteroides* and *Porphyromonadaceae_uc* decreased but *Cloacamonas_f_uc_g*, *GU454936_g*, *AC160630_g*, *Acholeplasmatales_uc_g* and *Mollicutes_uc_g* increased after 2 weeks storage of pig slurry (Fig 2). Differences in the dominant bacterial groups might stem from the environmental changes in pig slurry during the storage period. The pH is usually higher in stored manure (pH 8~10) than fresh manure (pH 6~7) [17]. Some strains of *Bacteroidetes* are detected with low abundance at increased pH [55]. Strains of the phylum *Cloacamonas_p* increased during waste treatment utilizing livestock manure [56]. These bacteria that use amino acids as an energy source could be increased because of the low levels of available carbohydrate for bacterial growth in stored manure [29]. PCA and PCoA score plots (Fig 4) support this hypothesis, showing that bacterial groups and odorous compound levels were changed after 2 weeks of storage.

Bacterial composition in pig slurry is correlated with the content of odorous compounds produced. In the current study, phenol, p-cresol, acetic acid, propionic acid and butyric acid displayed a strong positive correlation with *Bacteroides* and *Porphyromonadaceae_uc_g*. *Bacteroides* have previously been isolated from manure stored in deep pits [57, 58]. These bacterial species are able to secrete various enzymes and decompose a wide range of carbohydrates and proteins [59–61]. Among these enzymes, β-glucuronidase, which hydrolyses glucuronides to phenols and indoles, is produced at high levels [62]. In addition, hydrophobic molecules including phenols, indoles and their precursors could effectively transfer to the bacterial cell wall of *Bacteroides* [48, 63]. As a result, *Bacteroides* is related to changes in the levels of phenols and indoles in pig slurry. Most members of the family *Porphyromonadaceae*, including *Porphyromonadaceae_uc_g*, are saccharolytic excluding several proteolytic species [64–66]. Major end products of these strains are acetic, butyric, propionic and lactic acid, with iso-butyric acid as a minor product [64, 67–69]. Some strains are negatively related to indole formation [70].

Indole, skatole, iso-butyric acid and iso-valeric acid showed a positive correlation with *AC160630_g*, *Acholeplasmatales_uc_g*, *Mollicutes_uc_g* and *Cloacamonas_f_uc_g*. *Acholeplasmatales_uc_g* and *Mollicutes_uc_g* are members of the *Mollicutes* class in the phylum *Tenericutes*, Gram-positive and facultatively anaerobic [71]. The class *Mollicutes* arose from a *Clostridium* and descended from the *Streptococcus*/*Lactobacillus* group. *Clostridium* produces BCFAs and indoles from protein degradation [72, 73]. *Lactobacillus* strains convert indole-3-acetic acid, which is tryptophan metabolite formed by *Clostridium*, to skatole [42]. The *Mollicutes* class also produces some organic acids in anaerobic conditions. The members of the order *Acholeplasmatales* are the most abundant among the *Mollicutes* class and ferment glucose to produce acetic and lactic acid [74]. In addition, many strains of the *Acholeplasmatales* order are capable of synthesizing other fatty acids by utilizing acetate and butyrate [75]. *Mollicutes* strains are positively associated with the synthesis of valerate, iso-butyrate and iso-valerate, and can use amino acids as a carbon source [76]. The phylum *Cloacamonas_p*, including the genus *Cloacamonas_f_uc_g*, is a *Candidatus* taxon belonging to a subdominant bacterial group called WWE1 (Waste Water of Evry 1) and discovered by molecular inventories of an anaerobic digester [77]. WWE1 members could hydrolyze the cellulose via the release of extracellular cellulase. *Cloacamonas* spp. is syntrophic amino acid metabolizer, which could ferment a highly enriched hydrolysis product resulting from the hydrolytic activity of other bacteria [78] and produces fatty acids by phenol degradation [79]. Therefore, these bacterial strains are capable of the degradation of proteins for growth in the hypo-carbohydrate condition. In the present study, increased abundance of members of the phylum *Tenericutes* and *Cloacamonas* in stored slurry can be explain the protein availability for growth in the hypo-carbohydrate condition and then the high indoles and BCFA levels, which would be derived mainly from amino acid fermentation.

## Conclusions

Our results demonstrate that the storage period of pig slurry in a pit significantly affects the composition of odorous compounds produced as well as the bacterial community. Levels of odorous compounds were dramatically changed after 2 weeks of slurry storage. Phenols and SCFAs decreased, whereas indoles and BCFAs increased in the pig slurry. Accumulation of indoles and BCFAs is associated with increased pH during slurry storage and showed a strong positive correlation with members of the taxa *Tenericutes* and *Cloacamonas*. *Tenericutes* and *Cloacamonas* strains use proteins as a carbon source for growth and predominate in various environmental conditions. Our current study has significant value in identifying the causes of odor from pig houses. Based on our results, it is desirable that pig slurry needs to be discharged every 2 weeks to reduce the odor in pig house. Further investigation is necessary to control bacterial growth and identify fermentation patterns to increase the efficiency of odor reduction.
